# Network-based scoring system for genome-scale metabolic reconstructions

**DOI:** 10.1186/1752-0509-5-76

**Published:** 2011-05-19

**Authors:** M Ángeles Serrano, Francesc Sagués

**Affiliations:** 1 Departament de Química Física, Universitat de Barcelona, Martí i Franquès 1, Barcelona, 08028, Spain

## Abstract

**Background:**

Network reconstructions at the cell level are a major development in Systems Biology. However, we are far from fully exploiting its potentialities. Often, the incremental complexity of the pursued systems overrides experimental capabilities, or increasingly sophisticated protocols are underutilized to merely refine confidence levels of already established interactions. For metabolic networks, the currently employed confidence scoring system rates reactions discretely according to nested categories of experimental evidence or model-based likelihood.

**Results:**

Here, we propose a complementary network-based scoring system that exploits the statistical regularities of a metabolic network as a bipartite graph. As an illustration, we apply it to the metabolism of *Escherichia coli*. The model is adjusted to the observations to derive connection probabilities between individual metabolite-reaction pairs and, after validation, to assess the reliability of each reaction in probabilistic terms. This network-based scoring system uncovers very specific reactions that could be functionally or evolutionary important, identifies prominent experimental targets, and enables further confirmation of modeling results.

**Conclusions:**

We foresee a wide range of potential applications at different sub-cellular or supra-cellular levels of biological interactions given the natural bipartivity of many biological networks.

## Background

A crucial milestone to understand and control cellular behavior is the building up of reliable reconstructions of the interactions spanning different functional levels. Such reconstructions find a natural abstraction in the form of complex networks [1-3], where nodes represent cellular components such as genes, proteins, or metabolites, and edges identify the presence of biological interactions between them [4]. These network representations enable to map the large-scale structure of cellular interactions [5,6], to explore the basic principles of transcriptome and proteome organization [6], to identify missing genes encoding for specific metabolic functions [7], or to analyze emergent global phenomena in metabolism like robustness and regulation [8-11].

At present, the information for complex network representations of cellular systems comes primarily from web-based databases, oftentimes manually curated with information from multiple sources, like annotations from the literature or new experiments [12]. It is common to take the reliability of these data for granted and to draw from them resolute inferences about the properties or the behavior of the investigated organisms. Although, in general, observations are getting more and more precise, uncertainties about components or interactions remain [13,14]: experimental targets are many times biased towards the most rewarding in terms of expected impact, experimental evidence gathered with different methodologies is not always of the same quality, variability is unavoidably present in different organisms of the same species, and perfect environmental control is often difficult to achieve in experiments. In particular, high-throughput techniques produce massive data in comparison with more dedicated experiments at the price of repeatedly reported inaccuracy [15].

In this scenario, prediction of interactions in probabilistic terms is possible on the basis of the structure of the network alone and could serve to better characterize network-based descriptions of biological systems and as a guide for new experiments. In particular, the likelihood of biochemical reactions in cellular metabolisms can be assessed in terms of a scoring system that assigns a probability of occurrence to every reaction. This claims for a new interpretation, since canonically a chemical reaction either exists or does not. For network analysts, scoring a biochemical process in terms of its likelihood is a computational challenge. Starting from the assessment of individual links, as it has been done for instance for protein-protein interaction networks [16,17], the problem is conditioned by the need of non-local models that faithfully capture the large-scale statistical regularities of the networks, and by the requirement of sufficient reliability of the input observations. In this respect, the experimental reconstruction of genome-scale biochemical networks [18-21] is a well-established procedure and available data are of sufficient quality and can be exploited to get accurate network-based predictions.

Here, we propose a computational network-based reaction-scoring system and, subsequently, apply it to the metabolism of *E. coli*. We introduce a link prediction method that exploits the complex hierarchical structure and the statistical regularities of metabolic networks [22,23] and takes explicitly into account its bipartite nature [24,25]. Our model is adjusted to the observations in order to derive connection probabilities [26-28] between metabolite-reaction pairs and, after validation, we integrate individual link information to assess the likelihood of each reaction, both in absolute terms and relative to a random null model. Our network-based scoring system ranks reactions with a continuously distributed index which breaks the degeneracy and nested nature of experimental confidence scores [21]. However, the two schemes are differently targeted: confidence scores in the databases inform about the level of experimental evidence of a reaction, while the network-based score measures occurrence likelihood based on statistical inference. Our aim is, thus, to complement the existing experimental approach with new and essential network-based computationally inferred information.

## Results and Discussion

### Network-based reaction-scoring system

Beyond experimental evidence, it is possible to assess the likelihood of reactions in genome-scale metabolic reconstructions using theoretical models. Given an experimentally derived metabolic reconstruction, a score can be computed for each reaction on the basis of a suitable stochastic network-based model, as we propose in the next section. In the context of bipartite network representations [24,25], the model exploits the statistical regularities that underlie the structure of a metabolic network in order to ascertaining how well individual links between metabolites and reactions fit the observed topological patterns. In this way, it is possible to predict probabilities of connection for all potential interactions, those already present in the reconstruction and those absent. These probabilities are further integrated for the specific combination of metabolites involved in any particular reaction.

To define the network-based scores in quantitative terms, we interpret that a reaction is equivalent to the univocal combination of its associated metabolites, and that every metabolite *m *has a probability *p_mr _*of being associated to a reaction *r*. Once the connection probabilities between metabolites and reactions are estimated from the model, and assuming their mutual independence, the probability that a certain combination *ν *of metabolites co-occur in a reaction *r *is(1)

where the subindex *m *corresponds to metabolites in the predefined set *ν *and *m*' to those not included. The average number of co-occurrences can be calculated as the sum over all reactions(2)

A network-based reaction score can then be defined as the average number of occurrences *n_ν _*that the model associates to its particular combination of metabolites *ν*. Defined in this way, these scores break the degeneracy of reactions with identical experimental evidence.

### The Tree Distance Bipartite model

**Figure 1:**
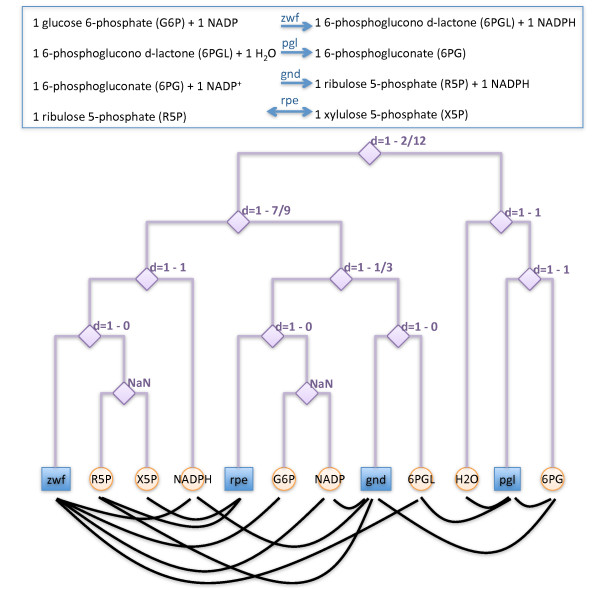
**Illustration of the method. Bipartite network representation and corresponding dendrogram**. We illustrate the model with four coupled stoichiometric equations in the pentose-phosphate pathway of *E. coli*. Reaction acronyms stand for the catalyzing enzyme: zwf, *glucose- 6- phosphate dehydrogenase *[EC 1. 1.1. 49]; pgl, *6- phospho-gluconolactonase *[EC 3. 1. 1.31]; gnd, *6- phosphogluconate dehydrogenase *[EC 1.1.1. 43]; rpe, ribulose- phosphate 3- *epimerase *[EC 5. 1.3. 1]. These equations are represented as an unweighted undirected bipartite network formed by connections (black lines) between reactions (blue squares) and metabolites (orange circles). Notice that metabolites or reactions are not connected among themselves. The model adjusts an underlying binary tree to the observed network structure where each internal node *t *is associated with a tree probability *ρ_t_*, that is transformed into a distance *d_t _*= 1 *ρ_t_*. Every pair reaction-metabolite with minimum common ancestor *t *is separated a distance *d_mr _*= *d_t _*(the NaN notation indicates that only one class of nodes populates the leaves of the child branches such that the corresponding internal node does not contribute).

In the following, we introduce and discuss a stochastic network-based model to estimate the connection probabilities between metabolites and reactions. Taking advantage of their natural bipartite nature, we consider network representations where metabolites and reactions appear as two different classes of nodes, and metabolites are connected by edges to the reactions they participate in. We consider the simplest unweighted undirected representation, where substrates and products are not differentiated. See Figure 1 for an example.

Previous works have shown that the complex organization of metabolic networks displays characteristic features shared by other complex networks: short topological diameter [29], steady state cycles [30] or structural robustness [11], for instance. We implement a large-scale model that takes advantage of some of those organizing principles, in particular the heterogeneity in the number of connections per metabolite (degree) [31], to infer connection probabilities *p_mr _*between metabolites and reactions. Network-based models are usually defined in terms of connection rules between the nodes. These laws are usually stated independently of observed systems to produce simulated networks that summarize their topological structure. Notice that here, in contrast, we compute the set of connection probabilities that has the maximum likelihood to reproduce the observed patterns in empirical metabolic networks, so we are solving the inverse problem.

Our first step is to assume a metric space underlying the structure of the empirical metabolic network. To this end, we fit to it a hierarchical random graph [27], once represented as a bipartite network with *M *metabolites and *R *reactions (see Figure 1). More specifically, we adjust the empirical bipartite network to a dendrogram, or binary tree structure, where metabolites and reactions appear as leafs. This tree represents the underlying metric space, and each of the *M *+ *R *- 1 internal nodes *t *in the dendrogram has an associated distance *d_t_*, so that each pair metabolite-reaction for which *t *is the lowest common ancestor is separated by a distance in the tree *d_mr _*= *d_t_*, independently from whether the link actually exists in the empirical network or not. We find these tree distances by fitting the tree to the empirical network data combining a maximum-likelihood approach with a Monte Carlo sampling method that explores the space of all possible dendrograms (see Methods). Our results are based on intensive numerical simulations that average a large number of samples in the stationary state when changes in the form of the dendrogram do not modify the likelihood function beyond fluctuations.

Once a distance *d_mr _*is associated to a metabolite-reaction pair, we correct for heterogeneity in the degrees of metabolites. We compute estimated connection probabilities *p_mr _*between all possible combinations metabolite-reaction as(3)

where *k_m _*is the degree of the metabolite and *μ *= 1/*R *ensures that network realizations with these connection probabilities have the same number of links as the observed network. From this equation, it is clear that the closer a pair is, the higher will be its connection probability. A Tree Distance Bipartite (TDB) score for every specific reaction can then be computed by applying Eq. (1) and Eq. (2).

### TDB scores for E. coli metabolism. Validation and Implementation

As an application of the methodology, we analyzed the *i*AF1260 version of the K12 MG1655 strain of *E. coli *[32] provided in the BiGG database [33,34]. From the empirical data, we built a bipartite network representation (see Methods and Additional File 1) with 1479 reactions and 976 metabolites (network provided in Additional File 2). As expected, the number of metabolites entering into a reaction, *k_r_*, follows a nearly homogeneous distribution with mean <*k_r _*> = 4.82 and mode 5. In contrast, the number *k_m _*of reactions in which a metabolite participates displays a heterogeneous degree distribution very close to a scale-free with  and an average degree <*k_m _*> = 7.30 (Additional File 1, Figure S2). The most highly connected substrates have more than a hundred and up to 841 connections (*h*^+^, *h*2*o*, *atp*, *pi*, *adp*, *ppi*, *nad*, *nadh*).

The TDB probabilities *p_mr _*were validated using standard tools in medical decision making and signal-detection theory [35], and compared with corresponding results from alternative models: the Configuration Model for bipartite networks [24,36,37] (CMB), the Hierarchical Random Graph model [27] generalized to bipartite networks (HRBG), and a local approach based on the computation of common neighbors (CN) [38] (reactions) between pairs of metabolites (see Methods). We checked two different aspects: the discrimination power of the models, and the behavior of the predictions under noisy conditions. We first measured the receiver operating characteristic (ROC) curve of predicted probabilities.

**Figure 2:**
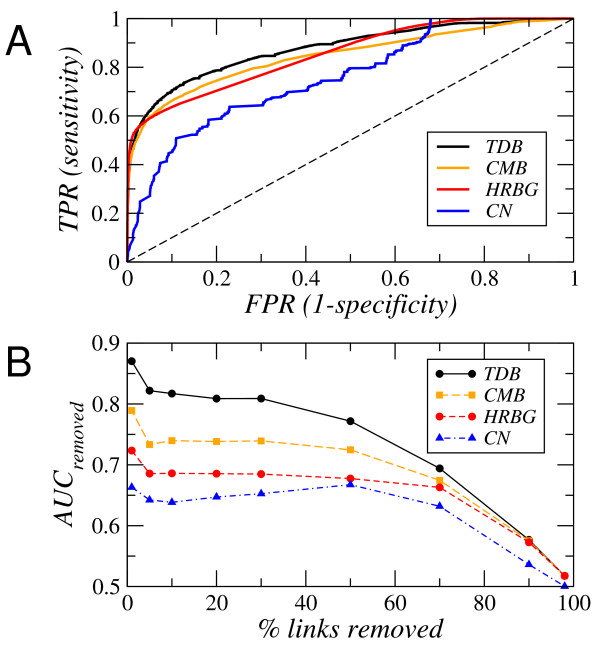
**Validation of the Tree Distance Bipartite model**. **A **Discrimination power as measured by the ROC curve: True Positive Rate (TPR) vs False Positive Rate (FPR). The TDB model is compared against the HRBG model, the CMB model and the CN model. **B **Response under noisy conditions. *AUC_removed _*is calculated as the probability that a randomly chosen removed connection has higher estimated connection probability than a randomly chosen unconnected reaction-metabolite pair.

To calculate the ROC curves we ranked the TDB probabilities from highest to lowest. We took every value in the rank as a threshold, and for each threshold we computed the fraction of true connections and the fraction of false connections above the threshold, the True Positive Rate (TPR) and the False Positive Rate (FPR) respectively, understanding that a true connection is an observed link in the empirical network and a false connection is an absent link. When representing the TPR in front of the FPR, a completely random guess would give a point along the diagonal line and points above the diagonal represent improved classification results. In relation to this, the area under the ROC curve (AUC) is a scalar measure of accuracy [39]. In the present context, it is calculated as the probability that a randomly chosen empirical link in the metabolic network has higher probability than a randomly chosen non-existing one. The ROC curves are shown in Figure 2A. As also corroborated by the AUC value (*AUC_TDB _*= 0.88, *AUC_HRBG _*= 0.85, *AUC_CMB _*= 0.85, and *AUC_CN _*= 0.76), the TDB model has more discriminatory power over a wide range of values as compared to other alternatives.

The second test helps to evaluate robustness against noise. Links were removed from the empirical network in order to see whether our algorithm was able to distinguish those from non-existing links. After removing a subset of links uniformly at random in the original network, a new set of connection probabilities was calculated on the basis of the remaining part of the network. The new probabilities associated to the removed connections were compared one by one to that of non-existing links. This statistic ranges from 0.5 to 1 and indicates how much better our method performs as compared to a by chance baseline accuracy of 0.5. We calculated this accuracy statistic, that is shown in Figure 2B, for different fractions of removed links. For the TDB model, when 1% of the 7127 links in the network are removed the index takes a value of 0.87, meaning that 87% of the times removed links are ranked higher in probability than non-existing links.

**Figure 3:**
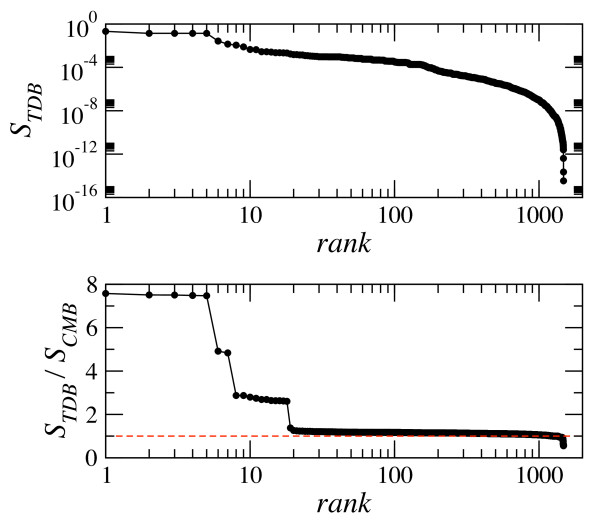
**TDB scores and comparison with CMB scores**. **Top**. The TDB scores, *S_TDB_*, have been ranked in decreasing order. The higher values form a short plateau and correspond to outlier values associated with reactions that only involve carrier metabolites (hubs). **Bottom**. Ratio of TDB scores to CMB scores ranked in decreasing order and compared to the baseline value equal to 1 (red dashed line). If ∑ = *S_TDB _*/*S_CMB _*> 1 (< 1) the network displays distance correlations (anticorrelations) which are absent in the random case.

In both tests, the TDB model outperforms all other strategies. Furthermore, TDB probabilities are well calibrated, meaning that the distributional forecasts and the observations are statistically consistent (Additional File 1, Figure S4). In view of these results, we accepted the accuracy at the statistical level of the predicted probabilities and we used them to compute theoretical scores  following Eq. (1) and Eq. (2) (provided in Additional File 2). Very high values of the TDB scores are typically associated to non-specific reactions dominated by carrier metabolites (hubs in network-based terms). At the top of the rank, the four different reactions with the highest values form a group of outliers (short plateau in the top graph of Figure 3) corresponding to reactions whose metabolites are exclusively hubs. These reactions are the *inorganic diphosphatase*, which hydrolyzes the pyrophosphate anion into inorganic phosphate; the *hydrolase of ATP into ADP*, which appears in the *Nucleotides Salvage *pathway and as a reaction for ATP maintenance requirement according to flux balance analysis, and mediates also the uptake of phosphate; and the *ATP synthase *in the *Oxidative Phosphorylation *pathway that takes four protons in periplasm to produce one ATP in cytosol from ADP. These non-specific reactions are at the end of catabolic chains and are likely to be shared by many different organisms. For instance, we checked that, according to the BiGG database [33,34], these reactions are also present in yeast (*S. cerevisiae i*MM904) and that *inorganic diphosphatase *and *ATP synthase *appear in human metabolism as well, where the *hydrolase of ATP into ADP *mediates the active transport of different metabolites across compartments. Even very simple organisms like *Mycoplasma pneumoniae *[20] use the *inorganic diphosphatase *reaction and the *hydrolase of ATP into ADP *in their metabolism. In [29], the presence of *inorganic diphosphatase *is reported in 101 out of 107 organisms spanning a wide taxonomical range, and the *hydrolase of ATP into ADP *is found into 25 of those. At the other extreme of the spectrum, low values of *S_TDB _*scores are associated with very specific reactions involving rare metabolites, in the sense that they enter a small number of reactions, or with unlikely reactions, like those involving a large number of metabolites. As an example, the *thiazole phosphate *synthesis is the largest reaction in the database involving twelve metabolites and has the lowest *S_TDB _*score, seventeen orders of magnitude below the maximum. In between, *S_TDB _*scores adopt a broad distribution of continuous values.

In the BiGG database, every reaction (except for exchanges and transports through outer membrane) is annotated with a confidence score assessing its level of experimental evidence. These values are discrete and range from 4 at the top, when there is direct biochemical proof, to 0 at the bottom, if the reaction is included with no experimental evidence but only because it improves modeling results. In between, values of 3 correspond to genomic evidence, level 2 refers to sequence homology evidence, and 1 stands for physiological evidence. This confidence scoring system presents some shortcomings, one being the degeneracy implicit in the use of only five discrete values for lists of hundreds or thousands of reactions, another the fact that different categories are not disjoint but nested, meaning that one backs the other. An additional worth remarking feature concerns the mean degree of the metabolites participating the reactions in each scoring level. Quite unexpectedly, we found a strong bias as this measure monotonously increases when decreasing rank from 4 to 1. More specifically, the average degree of metabolites entering into reactions with high level of confidence score is, on average, smaller than the average degree of metabolites associated to reactions with low level of confidence score.

It is not completely surprising, as the just mentioned test seems to suggest, that absolute indices based on the architecture of the empirical network may go differently as compared to qualitative indicators assigned from updated experimental information. We, thus, aim at complementing the potentialities of both scoring systems by cross-comparing them. In this respect, first we look for reactions with strong experimental evidence (values 4 and 3) that at the same time score low in the TDB system. Those correspond to very specific reactions that could be functionally or evolutionary important. Examples are the five *FMNH2-dependent monooxygenase *reactions in the *Inorganic Ion Transport *pathway, the *Pyridoxine *5'-*phosphate synthase *reaction in the *Cofactor and Prosthetic Group Biosynthesis *pathway, or the *Taurine dioxygenase *reaction in the *Alternate Carbon *metabolism. Conversely, a weak experimental evidence, scores 2 or 1 in the database, but a high value of the *S_TDB _*score, qualifies the reaction as a good target for further experimental verification in standard conditions. Many examples are found within the transport subsystems, like reactions of transport via ABC system (*iron (II) *and *(III)*, *phosphatidylglycerol*, *phosphatidate*). If the *S_TDB _*score is low, the reaction could be difficult to be observed experimentally except for very specific environments. Finally, high *S_TDB _*scores for reactions that where required for modeling, score 0 in the database, denote consistency between our model and steady-state flux optimization solutions. It is worth remarking that these reactions involve more than one carrier metabolite and stand out as potential experimental targets in standard conditions. However, a variety of reactions manifest discrepancies between both model-based likelihoods. More specifically, we differentiate two situations. The first does not entail contradiction and refers to very specific reactions with low *S_TDB _*scores involving rare metabolites that, on the other hand, seem to be essential for the viability of the bacteria according to flux balance analysis. This points out to potential experimental targets in non-standard conditions. Reactions in the subsystem of *Cofactor and Prosthetic Group Biosynthesis *are, for instance, in this category. In contrast, the second situation involves reactions like those with the highest number of participating metabolites, which according to confidence scores in the database were included in the reconstruction on the basis of modeling reasons while our methodology predicts very low scores. For them, we believe that low *S_TDB _*scores point indeed to insufficient ne detail resolution in the database and we are suspicious that new experiments may show a split of the high degree reactions into a set of coupled lower degree ones.

### Relative TDB scores

Along with absolute scores, we also analyzed relative scores defined on the basis of the Configuration Model for bipartite networks (CMB) [24,36,37] (see Methods). The latter assumes the actual degree distributions for metabolites and reactions and it is otherwise maximally random in the assembly of connections. The *S_CMB _*score of a reaction represents its probability of occurrence according to the configuration model and we use this value to compute the relative score as the ratio ∑ = *S_TDB _*/*S_CMB _*(provided in Additional File 2). Since differences between both scores are mainly related to the consideration of tree distances between metabolites and reactions in the TDB model, a relative score ∑ = *S_TDB _*/*S_CMB _*> 1 (< 1) points to the presence of tree distance correlations (anticorrelations) in the bipartite network, which are absent in the random case. In other words, a ratio higher (smaller) than one for a certain reaction indicates that its metabolites have a tendency to aggregate (avoid each other) as compared to the random case.

The ranking of relative scores is shown in the bottom graph of Figure 3, where several clusters can be differentiated. The first three clusters appear in slightly tilted plateaus with levels well separated by appreciable jumps. Each of them is formed by a subgroup of reactions that, according to the database, tend to belong to the same subsystem and share characteristic combinations of metabolites. The first group includes the *FMNH2-dependent monooxygenase *reactions, mentioned above as highly specific, with *flavin mononucleotide *and *sulfite *as reactants. The two reactions in the second plateau belong to the *Glycerophospholipid Metabolism *subsystem and are the only two in the database associated to *acyl phosphatidylglycerol*. The third cluster gathers together the eleven reactions containing *2-Demethylmenaquinone 8*. It is remarkable that, in general, the reactions in these clusters have attached a high DB confidence score. Exceptions appear in the third cluster, where one reaction has an experimental evidence score less than 3 while four reactions are included for modeling reasons, so that they become interesting targets for further experimental verification. For the rest, most of the scores have values above but close to one and there are also over two hundred reactions with ratios below one. At the very tail, one finds a set of reactions that share the common characteristic of being those with the highest reaction degree and with weak or just modeling evidence. In particular, *thiazole phosphate synthesis *is the sole reaction involving twelve metabolites in the database and has the lowest relative score ∑ = 0.3, and noticeably also the lowest absolute *S_TDB _*score (its confidence score in the database is 2).

Relative scores conform better than absolute scores to the idea of pathways as functional modules, since they accentuate the e ect of tree distances that we expect to be related with the modular organization of the network. This topic will be explored in depth in future work.

## Conclusions

The computational network-based TDB scoring system is able to assess, in probabilistic terms, the likelihood of reactions in metabolic reconstructions solely on the basis of the structure of the bipartite interactions between metabolites and reactions. It relies on a link prediction method adjusted to the observations that exploits the statistical regularities of the empirical network to estimate connection probabilities, that afterwards are integrated at the level of reactions. As a result, our TDB scoring system is able to break the degeneracy of currently employed scores that only use a discrete number of integers to label different levels of empirical evidence or model-based likelihood. We stress the main advantages of our procedure. First, our system permits to single out those reactions, well confirmed experimentally, that turn out to be highly specific as a potential signature of functional or evolutionary impact. Conversely, our analysis can help experimentalists to choose, among their potential targets, those reactions that appear, according to our scheme, more probable as compared to metabolic processes more prone to operate under non-standard conditions. In addition, our scoring system may provide information for unranked databases, and can be contrasted with other model-based alternatives such as steady state flux optimization solutions. When compared with a random null model that just accounts for heterogeneity in the number of connections per element, relative scores detect and quantify the tendency of groups of metabolites to aggregate or disaggregate. These distance-based correlations or anticorrelations in the underlying tree metric space raises a question worth exploring in the future in relation to functional modules.

In a broader context, many biological interactions find a natural representation in the form of bipartite networks. The ubiquity of these bipartite structures in cellular networks foretells a wide range of potential applications of the present methodology, from the estimation of gene-reaction associations in metabolic network reconstructions to the assessment of codon-gene association probabilities and of protein complexes.

## Methods

### Fitting the binary tree to the bipartite network

We adjust a binary tree to the observed data. The tree has *M *+ *R *- 1 internal nodes at its bifurcation points and *M *+ *R *leaves corresponding to the nodes of the metabolic bipartite network, *M *metabolites and its *R *reactions. Each internal node *t *has an associated tree probability *ρ_t _*that we transform into a distance *d_t _*= 1 - *ρ_t_*. Each pair metabolite-reaction having as lowest common ancestor *t *is then separated by a distance *d_mr _*= *d_t_*. Given a dendrogram, the internal probabilities *ρ_t _*are only dependent on the structure of the empirical bipartite network and can be calculated as the fraction of observed connections between leaves in each branch of the internal node over the total possible. To find the dendrogram that best fits the real data in terms of likelihood, we assume that all trees are a priori equally probable and explore the space of possibilities using a Markov chain Monte Carlo method [40] combined with a maximum likelihood approach, following the methodology in [27].

In statistical inference, the likelihood ℒ of a statistical model for a certain set of observed data is the probability that the model is a correct explanation, and allows us to estimate its unknown parameters. For a set of connection probabilities *ρ_t_*, and taking into account the underlying tree, the likelihood function becomes(4)

As for unipartite graphs, the variable *E_t _*stands for the number of actual edges in the empirical bigraph, in our case those that connect metabolites and reactions in the bipartite graph with *t *as their lowest common ancestor in the tree. The variable *ε_t _*corresponds to the total possible number of such edges given reactions and metabolites in the different branches of the common ancestor *t*, discounting internal combinations. In the unipartite case, *ε_t _*= *L_t_R_t_*, being *L_t _*and *R_t _*the number of leaves in the left and right subtrees rooted at *t*. In our scheme for bipartite networks, *ε_t _*= *L_tm_R_tr _*+ *L_tr_R_tm_*, counting possible combinations between metabolite leaves in the left subtree and reaction leaves in the right subtrees and vice versa. Then, *ρ_t _*= *E_t_*/*ε_t _*maximize ℒ.

Starting from a random configuration, we move among all possible sets of dendrograms by performing random swaps between one of the branches of a randomly chosen internal node and the alternative branch at its father level. This exploration is appropriate because it is ergodic and fulfils detailed balance. The likelihood of the new dendrogram produced in this way is computed and the dendrogram is accepted or rejected according to the standard Metropolis-Hastings rule [40]: the transition is accepted whenever the likelihood does not decrease and otherwise it is accepted with a probability exp(Δ*log*ℒ) (for computational purposes, it is more convenient to work with the logarithm of the likelihood function).

After a transient period when ℒ reaches its equilibrium value (except typical fluctuations) the system reaches a stationary state where we sample over 10^3 ^dendrograms at regular intervals to produce an average measure of *ρ_t _*and so of *ρ_mr _*for each possible metabolite-reaction pair. This model, the Hierarchical Random Bipartite Graph (HRBG), is a generalization for bipartite networks of the model introduced in [27]. As explained in the main text, in our TDB model we correct the tree distances *d_mr _*(*d_mr _*= 1 - *ρ_mr_*) for heterogeneity in the degrees of metabolites. We renormalize the distances according to Eq. (3) to produce the connection probabilities *p_mr _*that we use in the construction of the probabilistic reaction scores.

### Other alternative methods

#### The Configuration Model for bipartite networks

The Configuration Model for bipartite networks [24,36,37] (CMB) assumes a certain number of reactions *R*, a certain number of metabolites *M*, and their degree distributions *P*(*k_r_*) and *P *(*k_m_*), which should fulfill the requirement 〈*k_r_*〉 *R *= 〈*k_m_*〉 *M*, where 〈*k_r_*〉 is the average number of metabolites in the reactions and 〈*k_m_*〉 is the average number of reactions in which metabolites participate. Metabolites and reactions are partitioned into two different classes and each element in each class is assigned an expected degree from the corresponding distribution, which is attached in the form of stubs. Two stubs, one in each partition, are selected at random and the link between the metabolite and the reaction is created avoiding multiple connections. For the CMB model,  and Eq. (2) can be calculated analytically. Since the distribution of the bipartite degrees of the reactions is nearly homogeneous, *k_r _*≈ 〈*k_r_*〉, it becomes(5)

that gives the CMB probabilistic score for a metabolic reaction when its set of associated metabolites is *ν*.

#### Common Neighbors

Common neighbors [38] (CN) is a local similarity measure that counts the number of shared neighbors by a given pair. This measure represents a family of overlap measures quantifying similarity between nodes and a normalized version was specifically introduced for the study of the hierarchical modularity of metabolic networks and to delineate the functional modules based on the network topology [23]. In the case of bipartite metabolic networks, we define common neighbors for a pair of metabolites as the number of reactions in which they are concurrent, *o_mm'_*, and we estimate the probability of connection metabolite-reaction as .

### *E. coli *bipartite network representation

The *i*AF1260 version of the K12 MG1655 strain of *E. coli *[32] provided in the BiGG database [33,34] comprises 1039 metabolites and 2381 reactions of different nature happening in three different compartments: cytosol, periplasm, and a third symbolic one representing the extra-organism. For a meaningful conceptualization of this metabolic system as an undirected unweighted bipartite network, self-connections and dangling ends are avoided. To this end, we obviated exchange and diffusion reactions. We also neglected isomerizations and identified isomers as the same metabolite, but only whenever a reaction that carried out the isomerization could be identified in the database. This left a total of 1479 reactions that involve 976 metabolites. Finally, 5 metabolites were removed (*mn2*, *ca2*, *na1*, *ag*, and *cl*) because they do not enter in any reaction involving chemical transformation. See Additional File 1 for further details.

## Authors' contributions

All authors contributed equally to this work. Both authors have read and approved the final manuscript.

## Supplementary Material

Additional file 1 **Supporting figures and text for E. coli metabolism bipartite network representation and further validation of the model**. Click here for file

Additional file 2 **Database with reaction probability scores for E. coli metabolism and bipartite network representation in edge list format**. Click here for file
