# Supplementary material for: Network-based scoring system for genome-scale metabolic reconstructions
Source: BMC Syst Biol. 2011 May 19;5:76. doi: 10.1186/1752-0509-5-76 (PMC3113238; doi:10.1186/1752-0509-5-76)
Supplement: Additional file 1 — Supporting figures and text for E. coli metabolism bipartite network representation and further validation of the model. [file 1752-0509-5-76-S1.PDF]

Supporting Information
Network-based scoring system
for genome-scale metabolic reconstructions

M. Ángeles Serrano*, Francesc Sagués

Departament de Química Física, Universitat de Barcelona, Barcelona, Spain

∗ E-mail: marian.serrano@ub.edu

Contents

1 *E. coli* bipartite network representation 2

Supporting Figure S1 3

Supporting Table S1 5

Supporting Figure S2 6

Supporting Figure S3 7

2 Further validation of the model 7

Supporting Figure S4 8

1 *E. coli* bipartite network representation

In order to build a bipartite network representation of the metabolism of *E. coli*, we use the iAF1260 version of the K12 MG1655 strain provided in the BIGG database (http://bigg.ucsd.edu/). It comprises 1039 metabolites and 2381 reactions which include isomerizations, exchange reactions, intracompartment reactions, and different types of transport reactions (some of which involve chemical transformation) between three different compartments: cytosol, periplasm, and a third symbolic one representing the extra-organism. The most simple representation is in the form of an unweighted undirected network without self-loops or dangling ends (dead end reactions). More refined versions would take into account directionality of the reactions, stoichiometric coefficients, self-loops, etc..

Inside compartments, isomerization reactions (some reversible and some irreversible) transform the structure of one compound without altering its molecular formula. Isomers can have significantly different properties, so in principle it seems reasonable to consider those reactions in a network representation, and the two compounds as separate. However, the topological confidence score of those reactions will depend exclusively on the joint probability of occurrence of the pair of isomers and, most probably, the score would be expected to be low since they only enter together into those reactions. Our option (to be consistent with the treatment of diffusion reactions that lead to the same problematic as we explain below) is to neglect those reactions and to take the isomers as a single entity, whenever a reaction that carries out the isomerization can be identified in the database.

Exchange reactions represent the exchange of metabolites between the cell and the environment. These are reversible and involve a single metabolite. In a bipartite representation, exchange reactions would be dangling ends with a single incoming connection. Sinks needed to allow metabolites to leave the system (irreversible reactions in the cytoplasm) can be treated in the same way. Since they would count in the total amount of reactions but never two metabolites would enter them simultaneously, it seems reasonable to neglect them in the context of this work. Furthermore, exchanges or sinks have no associated reconstruction confidence score in the database.

Regarding transport reactions, there are several options. In *E. coli*, three different compartments are differentiated: cytosol, periplasm, and extra-organism. Every compound in a different compartment is considered as an individual specie and transport steps are formally considered as reactions transferring the compound belonging to one compartment into the same compound belonging to the other compartment (the respective concentrations can be different, and the compartments usually have different volume). Transport reactions between compartments are of different kind. Basic general mechanisms are:

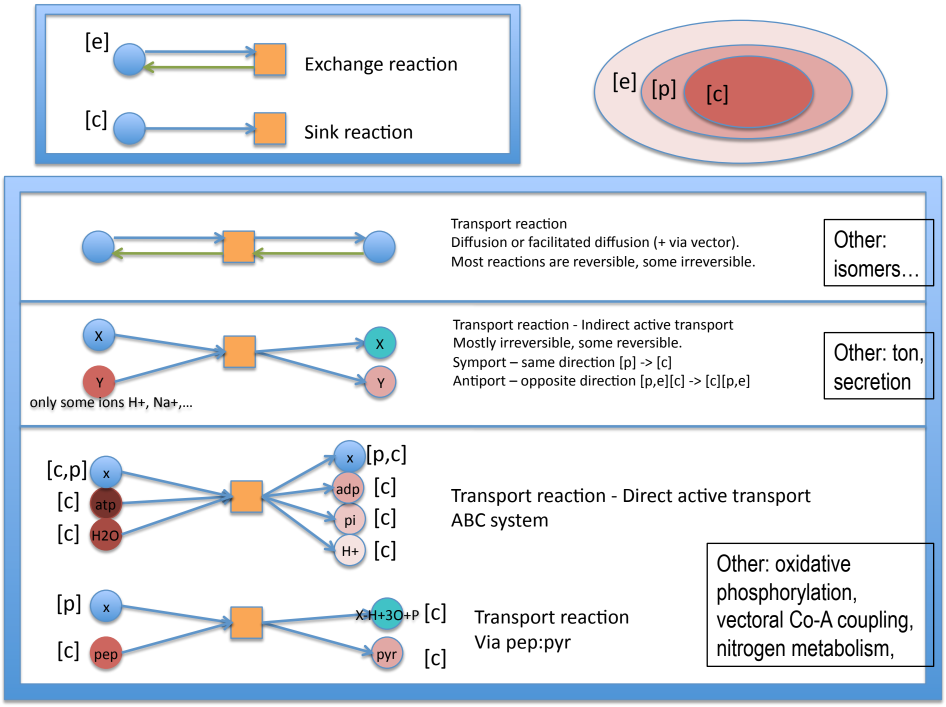

Supporting Figure S1: Some type of reactions and its topological bipartite representation in E. Coli metabolic network.

- Via diffusion (membrane permeable to water molecules and a few other small, uncharged, molecules like oxygen and carbon dioxide that diffuse freely in and out of the cell) or via facilitated diffusion (transmembrane proteins create a water-filled pore through which ions and some small hydrophilic molecules can pass by diffusion. The channels can be opened or closed according to the needs of the cell). Molecules and ions move spontaneously down their concentration gradient. No chemical transformation is involved. In the database, most are reversible processes while some are irreversible.

- Via active transport. Active transport is defined as mass transport from a region of lower to a region of a higher electrochemical potential. It is transport that requires energy. Active transport across biological membranes occurs via enzymes. Transmembrane proteins, called transporters, use the energy of ATP (hydrolyzation of ATP, in general $ATP + H2O- > ADP + Pi$, $\Delta G^0_{ATP} = -30,3KJ/mol$ or $-7.3kcal/mol$, enough to pump 2 sodium ions) to force ions or small molecules through the membrane against their concentration gradient. Active transport can be direct or indirect:

- Direct active transport. Some transporters bind ATP directly and use the energy of its hydrolysis to drive active transport (ATP powered pumps). In the database, these are named as ABC (ATP-Binding Cassette) transmembrane proteins, which expose a ligand-binding domain usually restricted to a single type of molecule at one surface and an ATP-binding domain at the other surface; the ATP bound to its domain provides the energy to pump the ligand across the membrane. In the database, hydrolyzation of ATP correspods to the chemical transformation $atp[c] + h2o[c]- > adp[c] + h[c] + pi[c]$ (cytoplasm, periplasm).
- Indirect active transport. A discrete class of proteins import or export ions and small molecules, such as glucose and amino acids, against a concentration gradient. These proteins use the energy stored in the electrochemical gradient of a directly-pumped ion to power the uphill movement of another substance, a process called cotransport. Direct active transport of the ion establishes a concentration gradient. When this is relieved by facilitated diffusion, the energy released can be harnessed to the pumping of some other ion or molecule. Symport pumps: the driving cotransported ion (H+,Na+) and the transported molecule pass through the membrane pump in the same direction. The driving ions flow down their concentration gradient while the coupled molecules are pumped up theirs; later the ion is pumped back out of the cell by a direct active transport process. Antiport pumps: the driving ion (again, usually sodium, proton/phosphate/succinate/nitrite/) diffuses through the pump in one direction providing the energy for the active transport of some other molecule or ion in the opposite direction. In any case, no direct chemical change in the database.

In the database, other transport reactions also appear. Some do not involve chemical transformation like for indirect active transport: via ton system, or secretion (without known transport mechanism). A second class involve chemical transformation of metabolites along transport: pep-pyr system, oxidative phosphorylation, vectorial Co-A coupling, nitrogen metabolism, etc..

In relation to the bipartite network representation, one needs to differentiate the different transport mechanism: diffusion, transport without chemical transformation, and transport involving chemical transformation of carriers (see Supp. Fig. S1).

Diffusion reactions do not transform any compound and involve the same metabolite both as input and output (self-loops in a metabolite one-mode projection), and so require some specific treatment. One possibility is to consider the metabolites in both parts of the reaction as different entities, the effective

pair. Since this is the only pair entering such reactions, the topological score is expected to be low as for isomerization reactions. However, since the metabolite is the same with the same properties, a better option in this case is to neglect these reactions and do not differentiate the metabolite in different compartments.

Other transport reactions without chemical transformation involve more than one metabolite, usually two that change compartment. In topological terms, this is a situation analogous to diffusion and the same treatment of ignoring the reaction and taking the metabolite in different compartments as the same can be applied.

Supporting Table S1: Classification of reactions in the database according to compartment implications, function and topological structure. The total number of reactions R in the database is 2381.

Classification of reactions in E. Coli metabolic network

Type	ID	Description	Number of reactions	Included in graph
internal	1	cytosol	1100	Yes
	2	isomerization in cytoplasm	59	No
	3	periplasm	190	Yes
	4	isomerization in periplasm	2	No
	5	extra-organism	8	Yes
exchange	6	sinks in cytosol	5	No
	7	exchange in extra-organism	299	No
transport	8	diffusion, facilitated diffusion, via vector, channel, flipping	346	No
	9	ABC system, direct active transport (+1 detoxification)	124	Yes
	10	symport, +1 similar reaction ID 11690	124	No
	11	antiport, +6 similar reactions Ids 11407, 11409, 11411, 11413, 11415, 11835	50	No
	12	ton system	11	No
	13	secretion (transport mechanism not known)	6	No
	14	reactions with transformation involving different compartments (pep:pyr, oxidative phosphorylation, vectoral Co-A coupling, nitrogen metabolism, etc.)	57	Yes

Transport reactions that need to transform metabolites to transfer a compound have different metabolites entering and leaving the reaction except the compound that is transported across compartments. To be consistent with the previous treatments, that metabolite in different compartments would be treated as a single entity.

Finally, chemical transformation reactions that happen in more than one compartment (or that need some component of a different compartment from where it is happening) can be treated as chemical

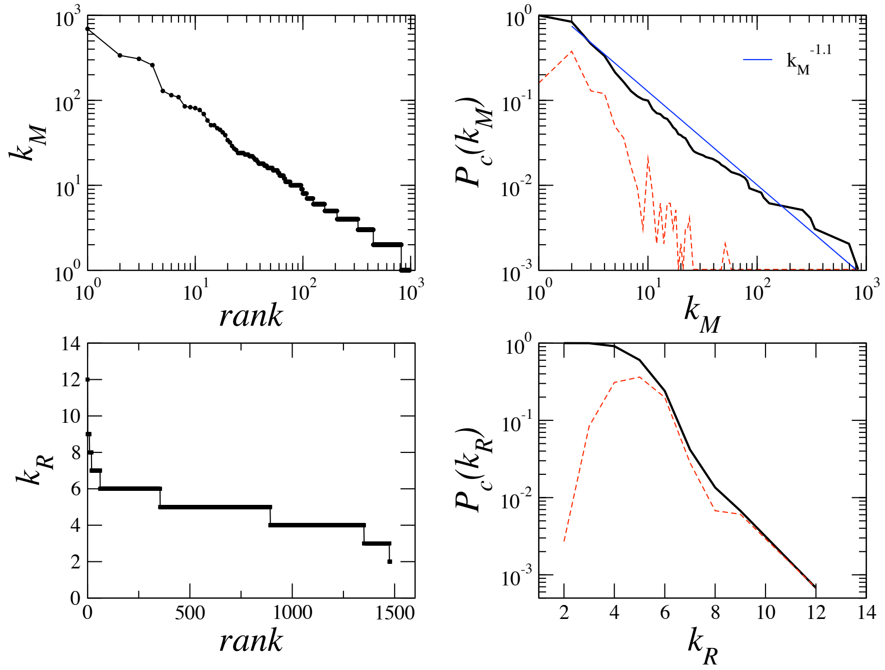

Supporting Figure S2: Rank distribution of degrees of metabolites and reactions and cumulative bipartite degree distributions. The degree of a metabolite is taken as the number of reactions it participates in and the degree of a reaction is the number of different metabolites it involves.

transformation reactions inside compartments.

In summary (see Supp. Table S1), we take metabolites as the same entity independently of the compartment and we obviate isomerizations, exchange, and diffusion reactions. This leaves a total of 1479 reactions out of 2381 that involve 976 metabolites out of 1039 (isomers have been identified with the same id). Apart from the identification of isomers, 5 metabolites have been removed ($mn2$ (id 30), $ca2$ (id 60), $na1$ (id 383), ag (id 651), and cl (id 654)) because they do not enter in any transformation reaction. They seem to be necessary to transfer compounds (typically h but also others) across compartments.

In Supp. Fig. S2, we show the cumulative bipartite degree distribution both for metabolites and reactions. The degree of a metabolite is taken as the number of reactions it participates in and the degree of a reaction is the number of different metabolites it involves. The number of metabolites entering into a reaction k_r follows a homogeneous distribution with mean $< k_r >= 4.82$ and mode 5. In contrast, the number k_m of reactions in which a metabolite participates displays a scale free degree distribution $P(k_m) \sim k_m^{-2.1}$ with an average degree $< k_m >= 7.30$. Currency metabolites are the most connected substrates, some with more than a hundred and up to 841 connections (h^+, $h2o$, atp, pi, adp, ppi, nad, $nadh$).

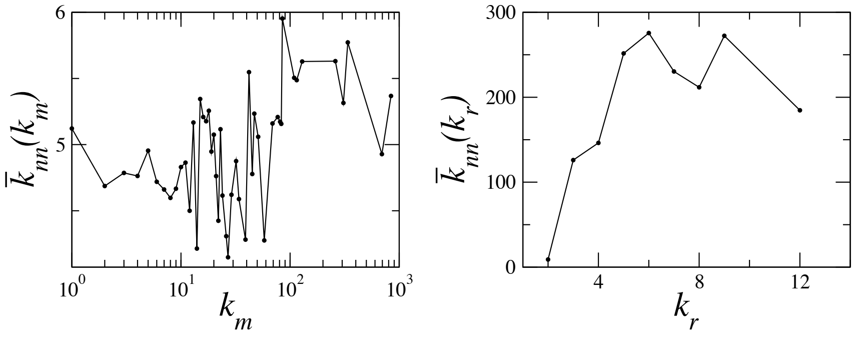

Supporting Figure S3: Average nearest neighbors bipartite degree of metabolites and reactions.

The average nearest neighbors bipartite degree of metabolites and reactions is displayed in Supp. Fig. S3. The rising of the average levels for higher values of the degrees means that the reactions involving more metabolites also involve more carrier metabolites (hubs). compartments and also enter in exchange reactions.

2 Further validation of the model

We provide here one more tests that probe the TDB method. To determine whether the TDB metabolite-reaction probabilities are calibrated, that is whether there is statistical consistency between the distributional forecasts and the observations, we perform a simple visual test shown in Supp. Fig. S4. We divide the range of p_{mr} into small bins such that each bin contains the same number of metabolite-reaction edges whose probabilities falls into that bin, and for each bin we calculate what fraction of those edges actually exists. As can be observed in the graph, these values follow a straight line up the main diagonal of the unit square when represented as a function of the average probability in the bins, meaning that the TDB probabilities are well calibrated. Notice that the original probabilities on the underlying tree in the TDB model, before being transformed into distances, are calibrated by definition (see Materials and methods in the main text of the manuscript). Their transformation into distances and the computation of the probabilities according to Eq.(3) (in the main text of the manuscript), which corrects for heterogeneity in the degrees of metabolites, do not alter appreciably the fact that events predicted to have a probability p actually happen a fraction p of the time.

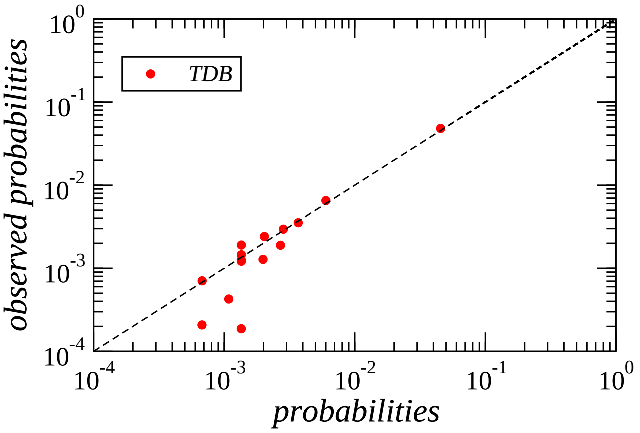

Supporting Figure S4: Validation of the Tree Distance Bipartite model. Analysis of calibration: agreement between the distributional probability forecasts and the observations.
